# Heritable Pulmonary Arterial Hypertension in a Patient With Empty Sella Syndrome: A Case Report

**DOI:** 10.7759/cureus.54632

**Published:** 2024-02-21

**Authors:** Bader Alghamdi, Shahad Aljuhani, Ghaday Alansari, Nouf M BinHumaid, Abdulkareem Alkahtani

**Affiliations:** 1 Pulmonology, King Abdulaziz Medical City, Jeddah, SAU; 2 College of Medicine, King Saud bin Abdulaziz University for Health Sciences, Jeddah, SAU; 3 Pulmonology, King Faisal Specialist Hospital & Research Centre, Jeddah, SAU; 4 Medical Imaging, King Abdulaziz Medical City, Jeddah, SAU

**Keywords:** non-bmpr2, heritable pah, genetics, empty sella syndrome, pulmonary arterial hypertension

## Abstract

Pulmonary arterial hypertension (PAH) is a progressive disease with multiple contributing factors. Genetics, epigenetics, hormonal, and immune factors all contribute to the development and progression of the disease. A number of endocrine disorders and metabolic syndromes are being studied for their potential role in the development of PAH. We report to you a case of a 32-year-old female with a rare presentation of a non-BMPR2 mutation heritable PAH complicated with empty sella syndrome and panhypopituitarism.

## Introduction

Heritable pulmonary arterial hypertension (HPAH) is considered rare and accounts for approximately 25% to 30% of idiopathic pulmonary arterial hypertension (IPAH) [1]. Most HPAH cases are caused by mutations in the BMPR2 gene (75%), while mutations in other genes are much less common (ACVRL1, BMPR1B, CAV1, ENG, SMAD9) [2]. However, in approximately 25% of families with HPAH, the mutation responsible has not been identified.

As part of the clinical assessment of HPAH, clinical findings must include confirmation of PAH (i.e., a mean pulmonary artery pressure at rest exceeding 20 mm Hg during cardiac catheterization) as well as the identification of a heterozygous pathogenic (or likely pathogenic) variant in a gene associated with HPAH and/or confirmation of PAH in the proband's family members [3].

The development of PAH is a multifactorial condition involving genetic, epigenetic, and immune-related factors. Several endocrine disorders and metabolic syndromes have been linked to the development of PAH, like thyroid disorders and their effect on the pulmonary vasculature. Moreover, there is new evidence showing a relationship between abnormal estrogen metabolism and the development of the disease, explaining the female predominance reported in the literature.

Empty sella syndrome (ESS) is a rare condition in which the subarachnoid space herniates into the sella turcica and compresses and flattens the pituitary gland. As a result of the compression of the pituitary parenchyma as well as the involvement of the pituitary stalk, hormonal deficiencies can occur to varying degrees [4].

As far as we are aware, this is the first reported case of a non-BMPR2 mutation HPAH in combination with partial ESS complicated by hypopituitarism.

## Case presentation

**Figure 1:**
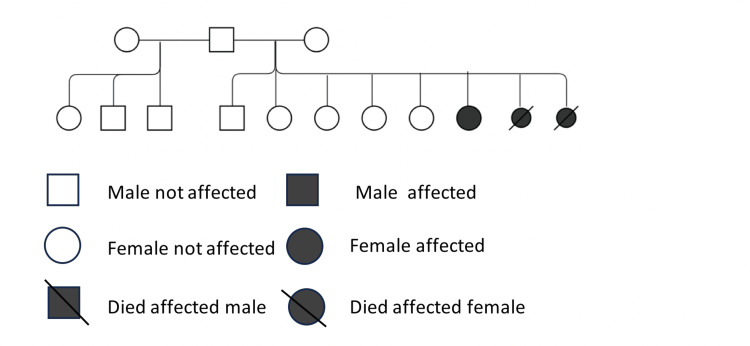
Family pedigree Representing family members affected by pulmonary arterial hypertension (PAH)

A 32-year-old female presented at the pulmonary hypertension clinic in 2015, complaining of shortness of breath (SOB)(New York Heart Association (NYHA) class I-II). She was diagnosed five years prior at another hospital with PAH and started on sildenafil 20 mg TID (three times a day) and bosentan 125 mg BID (twice a day). The patient denied experiencing any symptoms suggestive of right heart failure or chest pain, syncope, or pre-syncope. And there was no history of previous thromboembolic events. She had no history of cardiac or chronic lung diseases, and she did not report any symptoms of obstructive sleep apnea. However, she reported a history of menstrual cycle disturbances and revealed a family history of PAH. Two of her sisters were diagnosed with PAH based on a right heart catheterization. Both sisters passed away due to right ventricular failure at the age of 25 and post-RHC cardiac arrest at the age of 13, respectively. Both sisters had a history of menstrual irregularities but had not been investigated (Figure 1).

On physical examination, she had a short stature. There was no evidence of finger clubbing, jugular venous distension, or lower limb edema. Her chest was clear, and the cardiovascular examination showed S1+S2 with a loud P2.

Initially, in 2010 transthoracic ECHO for our patient at the age of 18 showed normal intracardiac anatomy and mild tricuspid regurgitation (TR) with right ventricular systolic pressure (RVSP) of 50 mmHg. Mildly dilated and hypertrophied right ventricle with no right ventricular outflow tract obstruction (RVOTO). No mitral regurgitation, aortic stenosis, aortic insufficiency, or patent ductus arteriosus (PDA). Normal aortic arch and good biventricular systolic function.

Her diagnostic right heart catheterization (RHC), which was done in November 2010 in another hospital, showed mean right atrial pressure (RAP) of 2 mmHg, right ventricle (RV) 52/-2 mean 4 mmHg, pulmonary artery pressure (PA) 49/21 mean 33 mmHg, pulmonary capillary wedge pressure (PCWP) mean of 5 mmHg and left ventricular end-diastolic pressure (LVEDP) of 5 mmHg. Cardiac output-Fick was 5.41 l/min and cardiac index was 3.7 l/min/sq m. Pulmonary vascular resistance (PVR) 5.1 WU.

As the patient established her care in our institution, a chest X-ray and CT were performed as an initial diagnostic workup in 2015. Both chest X-ray and CT demonstrated signs of PAH without parenchymal lung disease. A ventilation/perfusion (V/Q) scan showed no signs of chronic thromboembolic pulmonary hypertension. Her pulmonary function test revealed mixed obstructive and restrictive lung defects. This could be explained by PAH and bronchial asthma diagnoses.

After counseling, a genetic test was performed, showing no pathogenic variant associated with the BMPR2 gene. Further assessment of molecular genetic analysis of whole exome sequencing was done. This did not identify any clinically relevant variants with significant phenotypic overlap.

Given the patient's history of menstrual cycle irregularity and reported history of receiving growth hormone replacement therapy before, she was referred to the endocrine and obstetrics/gynecology (OB/GYN) services. She received the diagnosis of empty sella syndrome in addition to panhypopituitarism (adrenal insufficiency, central hypothyroidism, and central hypogonadism) (Table 1), and was placed on hormonal replacement therapy (levothyroxine 75 mg, hydrocortisone 10 mg AM, and 5mg PM, conjugated estrogen tablet 0.625mg daily and medroxyprogesterone 5mg for the first 12 days) with clinical and biochemical improvement at stable doses.

**Table 1:** Patient's pituitary hormone levels TSH: thyroid stimulating hormone; FSH: follicle-stimulating hormone; LH: luteinizing hormone; IGF-1: insulin-like growth factor 1

	Result	Reference range
TSH	3.23	0.6 - 5.8 mlU/L
Free T4	7.7	9 - 19 pmol/L
FSH	1.19	1.6 -15.3 lU/L
LH	0.17	1.3 - 35.2 lU/L
Prolactin	33.45	7.20 - 44.30 ug/L
Cortisol	<28	172 - 497 nmol/L
Estradiol	<37	73 - 1301 pmol/mL
IGF-1	14.88	85-283 ng/mL

In 2017, a repeated right heart catheterization was performed after three months (Table 2). Her six-minute walk test was 420 meters and brain natriuretic peptide (BNP) was 20 pg/Ml. She is a low-risk PAH on dual therapy with good compliance.

**Table 2:** Right heart catheterization measurements

Hemodynamic parameters	Obtained values	Normal values
Pulmonary artery pressure (mmHg) S/D/M (systolic/diastolic/mean)	61/25/39	15 to 25 / 8 to 15 / 10 to 20
Pulmonary capillary wedge pressure (mmHg)	13	less the 15
Right atrial pressure (mmHg)	Mean 5	0 to 8 mmHg
Right ventricle pressure (mmHg) S/D/M	59/1/11	20 to 30 / 3 to 7 / less than 33
Pulmonary vascular resistance (dynes-sec/cm^-5^) via Thermo method	495.24	30 to 180
Pulmonary vascular resistance (dynes-sec/cm^-5^) via Fick method	551.72	30 to 180
Stroke volume (ml/beat) via Thermo/Fick	60.00/53.83	50 to 100
Cardiac output (L/min) via Thermo/Fick	4.20/3.77	5 to 6
Cardiac index (L/min/m^2^) via Thermo/Fick	2.76/2.48	2.5 to 4.2

## Discussion

Heritable pulmonary arterial hypertension (HPAH) is a rare disease with several associated pathogenic mutations. It is essential to conduct a genetic test on patients with a history of familial PAH to identify the mutation responsible for the disease. This is important since specific mutations can alter the treatment and prognosis of the condition. It is usually the first step to perform a genetic test that consists of the most commonly known genetic predisposing mutations. If no mutation is identified, like in our patient, whole exome sequencing (WES) or whole genome sequencing (WGS) can be performed [3]. Considering that our patient had a clear family history of the disease, we performed a genetic test to determine if there was any pathological variant of the BMPR2 gene. The genetic test did not identify the presence of any known pathological mutation; similarly, the result of the second step, the WES test, was negative as well, so our patient's clinical phenotype was not explained by any mutation identified by the WES. However, around 25% of families with HPAH do not have any of the known mutations associated with the disease [5].

Similar to our patient, people with HPAH and BMPR2 mutation carriers are usually younger and present with a more severe clinical presentation at the time of diagnosis [6], and the mean age at diagnosis is 34.9±14.9 years [7]. The disease also affects women more than men [8], which is believed to be due to estrogen metabolism's crucial role in the development and progression of PAH.

In addition to HPAH, our patient also suffers from empty sella syndrome, a condition characterized by heterogeneous clinical manifestations and hormonal changes. She also suffers from hypopituitarism and is undergoing treatment for adrenal insufficiency, central hypothyroidism, and hypogonadotropic hypogonadism. 

A patient with HPAH who is also suffering from this condition is considered to have a rare presentation since we are not aware of any other instances in which this condition has been associated with HPAH. As thyroid disease has been associated with PAH for decades, both hypothyroidism and hyperthyroidism are commonly observed in patients with PAH [9]. Both increase cardiac output and pulmonary vascular resistance, which can lead to PH [10]. According to a retrospective study examining long-term survival in patients with hypothyroidism and IPAH, thyroid hormone replacement therapy was associated with favorable outcomes [11].

## Conclusions

Emerging evidence suggests a strong correlation between endocrine disorders and pulmonary arterial hypertension. The interplay between hormonal imbalances and the vascular system may contribute to the development and progression of PAH. Understanding this relationship is crucial for early detection, timely intervention, and improved treatment strategies for patients with both endocrine disorders and PAH. Further research is needed to elucidate the exact mechanisms and develop targeted therapies to address this complex association.
